# Evaluating classification tools for the prediction of *in-vitro* microbial pyruvate yield from organic carbon sources

**DOI:** 10.1371/journal.pone.0306987

**Published:** 2024-07-11

**Authors:** Manish Pant, Tanuja Pant

**Affiliations:** 1 IMS Engineering College, Ghaziabad, Uttar Pradesh, India; 2 Kumaun University, Nainital, Uttarakhand, India; University 20 Aout 1955 skikda, Algeria, ALGERIA

## Abstract

The laboratory-scale (*in-vitro*) microbial fermentation based on screening of process parameters (factors) and statistical validation of parameters (responses) using regression analysis. The recent trends have shifted from full factorial design towards more complex response surface methodology designs such as Box-Behnken design, Central Composite design. Apart from the optimisation methodologies, the listed designs are not flexible enough in deducing properties of parameters in terms of class variables. Machine learning algorithms have unique visualisations for the dataset presented with appropriate learning algorithms. The classification algorithms cannot be applied on all datasets and selection of classifier is essential in this regard. To resolve this issue, factor-response relationship needs to be evaluated as dataset and subsequent preprocessing could lead to appropriate results. The aim of the current study was to investigate the data-mining accuracy on the dataset developed using *in-vitro* pyruvate production using organic sources for the first time. The attributes were subjected to comparative classification on various classifiers and based on accuracy, multilayer perceptron (neural network algorithm) was selected as classifier. As per the results, the model showed significant results for prediction of classes and a good fit. The learning curve developed also showed the datasets converging and were linearly separable.

## Introduction

*In-vitro* experimental designs viz. screening designs (taguchi and plackett-burman), response surface methodology designs (Box-Behnken design and Central Composite design) have been used to estimate intrinsic yields for microbial production [1]. These operative designs have their own demerits including lack of diversified applications, rigid structure, statistically derived predictive results which leads incoherent results for natural phenomenon. The prediction and analysis of class properties is also very limited since very few models are applicable in this regard. Traditionally, artificial neural network (ANN) and genetic algorithms have predominantly been used for predictive models [2]. Recent state of art studies have shown improved classification yields, including gene selection using cuckoo search algorithm [3], deep learning classification of cancer disease [4], hybrid feature selection of cancer [5], marine predator chaotic algorithm [6], and hybrid cuckoo search with Harris Hawks optimization [7]. However, such frameworks are unable to determine performance of class variable (process variables) under typical nutrient limitations and complex bioprocessing conditions. On the other hand, machine learning tools qualitatively represent synergy towards predictive modelling through full factorial designs [8]. The use of *in-vitro* dataset for predictive modelling and performance efficacy evaluations have limitations including, lack of consistent data reports, varied response variables and process parameters and infrequent designs [9]. To resolve these issues, invariant full factorial designs and subsequent class evaluations can be performed to make sufficiently learnable machine learning classifier models.

Pyruvate (reduced pyruvic acid) is traditionally obtained using microbial production on inorganic media compositions [10]. The current study utilises *in-vitro* experimental dataset from organic based media composition for pyruvate production. The *in-vitro* experimental design includes factors (independent variables), constant parameters, and responses (dependent variables). The selection of class variable is based on critical factors to observe the influence on accuracy using applicable classifiers. The comparative experimental setup (varied nutrient sources) will help in evaluating the performance of classifier on separate sources. The aim of the study is to investigate the data-mining accuracy on the dataset developed using experimental production for the first time. The attributes were subjected to comparative classification on various classifiers and the most appropriate classifier was selected for the development of model.

## Materials and methods

### Classification

The full factorial design based glycerol dataset for comparison of classifiers was built in Microsoft Excel in the ARFF (Attribute-Relation File Format) as glycerol.arff file. The glycerol dataset was directly loaded in the pre-process tab of WEKA tool. The ARFF datasets for the two organic carbon sources (rice straw and jackfruit rind) were built and loaded independently in the pre-process tab. The attributes (reducing sugar and biomass concentration) were removed to deduce the classification of CSL concentration on pyruvate concentration. Moreover, CSL concentration, incubation time and incubation temperature were set to nominal and numeric values for pyruvate concentration. CSL concentration (nominal attribute) was pre-processed as the class variable for the classifier prediction of the numeric dataset (pyruvate concentration). The CSL concentration was selected as the class variable, since it acts as the replacement of inorganic nitrogen macro-nutrient in the media formulation. The processing conditions such as incubation time and incubation temperature are easily optimised in the fermenter. Moreover, the input variable in the fermenter such as inflow rate can be modelled as a class variable in real world problems associated with product yield. The response variables are results observed as the significant/non-significant values associated with the variations in input variables. Moreover, any changes in the independent variable provides a causal effect relationship on dependent variables [11]. The converse may also be true, thus the changes in responses can be monitored with changes in input variables and any combination thereof.

Mathematically,

f(a,b,c)=d;f(a,b,d)=c;f(a,c,d)=b;f(b,c,d)=a

Thus, utilisation of input variables as class variables can help in classification model development in the laboratory experiments. Moreover, the numbers of experimental runs are generally limited, and the utilisation of full factorial design is crucial in this regard.

### Comparison of classifiers

Classification uses the application of standard algorithms to clearly describe, distinguish discrete classes for the model and to predict each iteration in the dataset under experimental conditions [12]. However, the classification algorithms used to solve various problems is extremely diverse. Hence, it is imperative to study in order to deduce the most suitable classifier algorithm for application in the target problems [13]. In this study, all possible classifiers were investigated in terms of classifier accuracy using knowledge flow environment of WEKA tool. The statistical measures (highest correctly classified instances, ROC area, etc.) were used in the screening and selection of classifier for model development (S4 Fig in S1 File). During screening of classifiers and model development, the standard glycerol dataset was subjected to 10X cross validation to resolve the issues of overfitting. The classifier selected at this step was applied to the other carbon sources for classification of CSL concentration.

### Model validation

After screening of classifiers, the classifier with highest accuracy i.e. multilayer perceptron was used to develop model for the three carbon sources and the standard statistical measures are represented. The linear separability can be investigated for the classification of data using the learning curve (S5 Fig in S1 File). If the classification of data is plotted on a single line and the classes can be separated by a single point it is said to be linearly separable [14]. The learning curve for the screened classifier was built on percent incorrect with confidence level less than 5% (S1 Table in S1 File). Finally, the classifier was validated using margin curve, classifier errors and cost curve visualisations for the three datasets.

## Results and discussion

### Experimental summary

In the experimental procedure, Corn Steep Liquor (CSL) served as the nitrogen alternative in the culture medium, while two carbon sources, namely rice straw and jackfruit rind, were selected as alternatives to glycerol for comparing overall pyruvate yield [15]. The carbon sources (viz. glycerol, rice straw and jackfruit rind) based media formulations were investigated and compared on varied levels of processing parameters including, CSL concentration (%v/v), incubation time (h) and incubation temperature (°C) as independent variables in the full factorial design. A full factorial design is a rigorous experimental design employed in scientific research, particularly in the field of experimental design and statistical analysis. It systematically explores the effects of multiple independent variables on a dependent variable by examining all possible combinations of the levels of each independent variable. Mathematically, a full factorial design with k factors at l levels each can be represented by the equation:

N=l^k

N denotes the total number of experimental runs. This equation underscores the exponential growth in runs as the number of factors or levels increases. For instance, a 3-factor design with 2 levels per factor requires 2^3 = 8 runs, while a 4-factor design with 3 levels per factor demands a substantial 3^4 = 81 runs. The crux of a full factorial design lies in its ability to estimate model parameters. The linear regression model, a common choice, incorporates the following terms:

y_ij…n=μ+Σα_i+Σβ_j+Σγ_k+Σ(αβ)_ij+Σ(αγ)_ik+…+ε_ij…n

*y*_*ij*…*n*: Represents the response variable at the kth level of factor k, the jth level of factor j, and so on.

*μ*: Represents the overall mean response.

*α*_*i*, *β*_*j*, *γ*_*k*: Represent the main effects of factors i, j, and k, respectively.

(*αβ*)_*ij*: Represents the two-factor interaction effect between factors i and j.

(*αγ*)_*ik*: Represents the two-factor interaction effect between factors i and k.

*ε*_*ij*…*n*: Represents the random error term.

In this study, pyruvate concentration (g/L), reducing sugars (g/L), and biomass concentration (g/L) were considered as the dependent variables. All experimental runs were performed independently in triplicates and the average values were analysed. The equation for number of experiments required for each carbon source is

Numberofexperimentsforeachcarbonsource=5levels×3levels×3levels=45experiments

The primary purpose of a full factorial design is to systematically investigate and quantify the main effects of each independent variable, as well as the potential interactions between them. Main effects represent the influence of a single independent variable on the dependent variable, while interactions reveal whether the effects of one variable depend on the level of another. The estimation of these parameters allows for a comprehensive analysis of the individual and combined influences of the factors on the response variable. Main effects reveal the average change in the response due to a single factor. Interaction effects, however, capture how the effect of one factor depends on the level of another. The power of a full factorial design lies in its ability to provide a complete picture of the factor space, potentially revealing unexpected interactions and non-linear relationships. This comprehensive approach aids in elucidating complex relationships within a system, providing a detailed understanding of how multiple factors collectively contribute to the observed outcomes. Additionally, the design facilitates the identification of optimal conditions for the desired outcome, thereby enhancing the efficiency of experimental investigations. However, its resource-intensive nature and complex data analysis can pose challenges, particularly with high-dimensional designs. Consequently, careful consideration of factors, levels, and available resources is crucial before embarking on a full factorial experiment. The individual run in the experiments were considered as data points for the three datasets in each of the carbon sources, respectively (S2 Table in S1 File).

### Screening of classifiers

The unbiased estimate of a classifying model’s efficiency, the statistical measures have to be evaluated from the dataset used in the model building process [16]. Common statistical measures of classification are sensitivity, specificity, accuracy and the area under the ROC curve (or, equivalently, the c-index). These values are essential in the determination of a comparatively superior for model building process [17]. The class variable, CSL concentration (class value ‘1’) was used to investigate the accuracy of various applicable classifiers. The experimental design was screened and modelled with glycerol as standard carbon source. The classifier comparison was investigated for pyruvate concentration with results using *in-vitro* experimental data. The performance of each classifier was evaluated with statistical summary as shown in **Table 1** representing model performance chart (S1 Fig in S1 File). The classifier selected at this step was applied to the organic carbon sources for classification of CSL concentration. **Table 1** represents important statistical measures from the text viewer, such as correctly classified instances, incorrectly classified instances, Receiver Operating Characteristic (ROC) area, Precision Recall Curve (PRC) value and Cohen’s kappa value for the classifier algorithms. The PRC is a graphical representation of the trade-off between precision and recall across various decision thresholds. It evaluates a classification model performance in terms of positive predictive value (precision) and its ability to capture positive instances (recall) at different points along the curve. AUC-PRC quantifies the overall performance of a classification model by calculating the area under the PRC curve. A higher AUC-PRC value (closer to 1) indicates better precision-recall performance, while a value of 0.5 suggests performance equivalent to random chance. Cohen’s kappa statistic is a measure of inter-rater agreement that corrects for the possibility of chance agreement. The kappa statistic evaluates the level of agreement beyond what would be expected by chance alone. The value of kappa ranges from -1 to 1, with 1 indicating perfect agreement, 0 suggesting agreement equivalent to chance, and negative values implying agreement worse than chance. In technical terms, a higher kappa value reflects a higher degree of reliability in the raters’ judgments, while a lower value indicates a weaker level of agreement. It is used to assess the reliability of categorical data interpretations and helps in the understanding of agreement than simple percentage agreement metrics. The formula for Cohen’s kappa is expressed as

$$Cohen^{\prime }s\;Kappa\;Statistic\;(\kappa )=\frac{p_{0}-p_{e}}{1-p_{e}}=1-\frac{1-p_{0}}{1-p_{e}}$$

P_o_ is the relative observed agreement among raters and P_e_ is the hypothetical probability of chance agreement [18]. As shown, the highest accuracy corresponds to multilayer perceptron (68.8889%) using 10X cross validation fold-maker, followed by simple logistic (46.6667%) and the lowest for IBk (0%). It is shown that simple logistic regression predictive classifier is weaker than MLP in terms of data mining accuracy. Generally, both models perform on about the same level with the more flexible neural networks generally outperforming logistic regression [19]. In this experimental comparison, we can say that multilayer perceptron (MLP) was the best scheme in all applicable classifiers (as per ROC value results). Thus, multilayer perceptron (MLP) was selected as the classification algorithm for three carbon sources to create models and summary for predictive classification of datasets in the microbial production. The generic object editor of multilayer perceptron was built with hidden layers as wildcard value “a” (attributes + classes), learning rate as 0.3, momentum 0.2 and validation threshold 20 [20].

**Table 1:** Statistical summary of classifier algorithms on the dataset (glycerol).

Algorithms/Scheme	Statistics summary						
	Correctly classified instances	Incorrectly classified instances	Kappa value	Mean absolute error	RMSE	ROC area	PRC value
**KStar**	3 (6.6667%)	42 (93.3333%)	-0.1667	0.3408	0.4566	0.420	0.199
**Multilayer perceptron**	31 (68.8889%)	14 (31.1111%)	0.6111	0.1898	0.3062	0.891	0.728
**REPTree**	13 (28.8889%)	32 (71.1111%)	0.1111	0.2962	0.3999	0.575	0.289
**BayesNet**	1 (2.2222%)	44 (97.7778%)	-0.2222	0.3381	0.4313	0.233	0.167
**IBk**	0 (0%)	45 (100%)	-0.25	0.3912	0.5983	0.375	0.185
**RandomForest**	3 (6.6667%)	42 (93.3333%)	-0.1667	0.3393	0.4647	0.448	0.230
**SimpleLogistic**	21 (46.6667%)	24 (53.3333%)	0.3333	0.2254	0.3383	0.817	0.565
**SMO**	1 (2.2222%)	44 (97.7778%)	-0.2222	0.3644	0.4713	0.211	0.160
**J48**	8 (17.7778%)	37 (82.2222%)	-0.0278	0.3077	0.447	0.534	0.240
**DecisionStump**	13 (28.8889%)	32 (71.1111%)	0.1111	0.2869	0.3817	0.557	0.265
**RandomTree**	11 (24.4444%)	34 (75.5556%)	0.0556	0.3022	0.5497	0.528	0.253
**ZeroR**	5 (11.1111%)	40 (88.8889%)	-0.1111	0.3208	0.401	0.414	0.185
**PART**	7 (15.5556%)	38 (84.4444%)	-0.0556	0.3173	0.4572	0.503	0.219
**OneR**	12 (26.6667%)	33 (73.3333%)	0.0833	0.2933	0.5416	0.542	0.220
**DecisionTable**	4 (8.8889%)	41 (91.1111%)	-0.1389	0.3208	0.4506	0.468	0.214
**JRip**	11 (24.4444%)	34 (75.5556%)	0.0556	0.2893	0.3992	0.553	0.297
**LWL**	3 (6.6667%)	42 (93.3333%)	-0.1667	0.3153	0.4241	0.464	0.232

### Multilayer perceptron

Multilayer Perceptron (MLP) neural networks are formidable classifiers, showcasing adaptability to complex patterns and non-linear relationships in diverse datasets. The hierarchical architecture allows for the automatic extraction of intricate features and representations, with applications in image recognition, natural language processing, and various other domains. It consists of a simple system of artificial neurons connected by weights and output signals, which are a function of the sum of inputs for the modified neuron from a linear activation function [21] and is described as.


$$y(v_{i})=\tanh\left(v_{i}\right)\;and\;y(v_{i})={\left(1-e^{-v_{i}}\right)}^{-1}$$

The hyperbolic tangent function ranges from -1 to 1, and the logistic function is similar in shape but ranges from 0 to 1. Here y_i_ is the output of the i^th^ node (neuron) and v_i_ is the weighted sum of the input connections. The network is divided into three layers: input, hidden, and output layer. The input layer receives the value vector for network initialization, the hidden layer performs training, and the output layer receives the output vector [22]. The main adjustable parameters are the maximum amount of iterations, learning rate, momentum, and the number of neurons in the hidden layer [23]. However, there are major limitations including, computational complexity, prone to overfitting, hyperparameters sensitivity, and the data preprocessing requirements. Training deep architectures requires substantial labelled data, and in scenarios with limited data, overfitting becomes a concern, needing careful regularization. The computational complexity of deep MLPs, especially with numerous parameters, requires significant computational resources and time. Sensitivity to hyperparameters poses another challenge, and requires meticulous tuning for optimal performance. Additionally, the model’s performance is dependent on proper preprocessing and could be adversely affected by the scale and distribution of input features. Alternative models or hybrid approaches could also be applied depending on the specific characteristics and requirements of the real-world datasets and their applications. In this study, the datasets from all the carbon sources were modelled, measured and analysed with training set validation under multilayer perceptron from the classifier tab. The graphic user interface with intermittent weighted functional nodes in multilayer perceptron provides model summary, accuracy by class and confusion matrix developed under multilayer perceptron and the statistical measures for all carbon sources [24].

The application of classifier model on the three carbon sources is performed on the basis of correctly classified instances, Cohen’s Kappa statistic, Root mean squared error, Precision, Recall, and ROC area as shown in **Table 2**. Correctly classified instances are used to evaluate the model performance in correctly assigning instances to their respective classes with reliability, model generalisation and cost reduction. The design model summary **(Table 2)** for glycerol, rice straw and jackfruit rind showed significant results with correctly classified instances as 93.34%, 91.12% and 97.78% respectively (S3 Fig in S1 File). Cohen’s Kappa statistic addresses issues of chance agreement, evaluates model performance, and contributes to the reliability and validity of classification outcomes. The Cohen’s kappa statistic value also showed strong (0.8–0.9) to almost perfect (above 0.9) results as 0.9167, 0.8859 and 0.9722 respectively. RMSE measures the average magnitude of the errors between predicted and actual values, providing a comprehensive view of the model’s accuracy. For a model to be considered a good fit, the values of root mean squared error should be less than 0.5 [25]. For the three carbon sources, the values of root mean squared error (RMSE) were 0.2252, 0.2103 and 0.1423, respectively. In a similar study, the classifier prediction of activation energy in biomass wastes yielded multilayer perceptron with RMSE value of 0.2348 [26]. Multilayer perceptron when used for optimising spray drying process (yield %) yielded RMSE value of 1.98 as compared to RSM value of 2.44 [27]. Thus, the classifier model developed for the three carbon sources is significant, a robust measure for inter-rater reliability of attributes, and is able to predict the data accurately [28]. To investigate the overall accuracy of the model, detailed accuracy by class is observed using precision, recall and ROC area value [29]. The ROC curve is a graphical representation of the trade-off between the true positive rate (sensitivity) and the false positive rate (1-specificity) across various threshold values. AUC-ROC quantifies the overall performance of a classification model by calculating the area under the ROC curve. A higher AUC-ROC value (closer to 1) indicates better discrimination performance, while a value of 0.5 suggests performance equivalent to random chance. It condenses the information from the entire ROC curve into a single value. For the model, to predict accurately ROC area value 0.7–0.8 is considered acceptable, 0.8–0.9 is excellent and greater than 0.9 is considered outstanding [30]. The results for detailed accuracy by class for the three carbon sources show significant results for multilayer perceptron (S3–S5 Tables in S1 File). The weighted average value of ROC area for glycerol, rice straw and jackfruit rind is 0.978, 0.967 and 0.994, respectively. The high value of ROC area (> 0.9) represents the model for the prediction of classes in CSL concentration with variable pyruvate concentration from the dataset obtained experimentally [31]. Hence, it can be considered appropriate in terms of accuracy for the respective classes in the dataset. Precision and recall provide insights into different aspects of a model’s performance including, when dealing with imbalanced datasets or when different costs are associated with false positives and false negatives. Similarly, weighted average results for precision and recall for the three carbon sources were 0.944 and 0.933, 0.918 and 0.911, and 0.980 and 0.978, respectively. The applications of multilayer perceptron have been limited to modeling, prediction and optimisation of process parameters of physico-mechanical process or medical applications. The current study entails the application to *in-vitro* studies for the prediction of class variable within the experimental dataset. Hence, the model showed an accreditation of multilayer perceptron in practical applications for full factorial experimental design. The training set modelled multilayer perceptron presented the confusion matrix with high accuracy and low error (S6–S8 Tables in S1 File).

**Table 2:** Model summary developed under multilayer perceptron.

	Glycerol	Rice straw	Jackfruit rind
Correctly classified instances	93.34	91.12	97.78
Cohen’s Kappa statistic	0.9167	0.8889	0.9722
Root mean squared error	0.2252	0.2103	0.1424
Precision	0.944	0.918	0.980
Recall	0.933	0.911	0.978
ROC area	0.978	0.967	0.994

### Model validation

**Fig 1:**
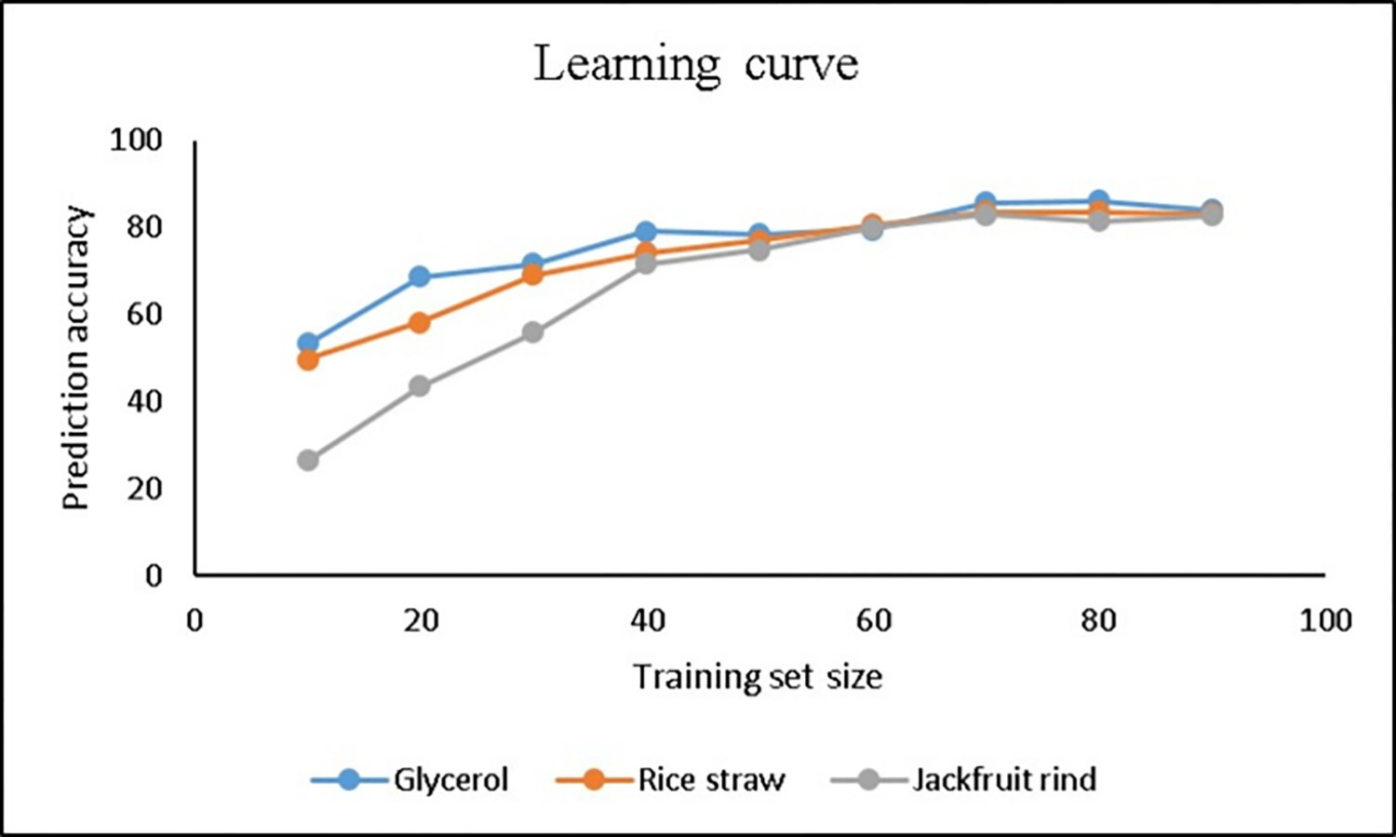
Learning curve for the three carbon sources.

The learning curve (training set size vs prediction accuracy) represents the rate of performance of the model when a percentage of data is removed from the dataset [32]. In contrast, ROC curve does not show learning instead it shows performance of the overall model [33]. Thus, to understand the ability of a model to learn the effect of attributes of dataset, learning curve is an essential constraint. For a model to be a good fit, the goal of the learning algorithm is to exist between an under fit and over fit. A good fit is identified by a training and validation loss that decreases to a point of stability with a minimal gap between the two final loss values [34]. The datasets from the three carbon sources were used in investigating the learning curve for the classifier and is shown in **Fig 1.**

**Fig 2:**
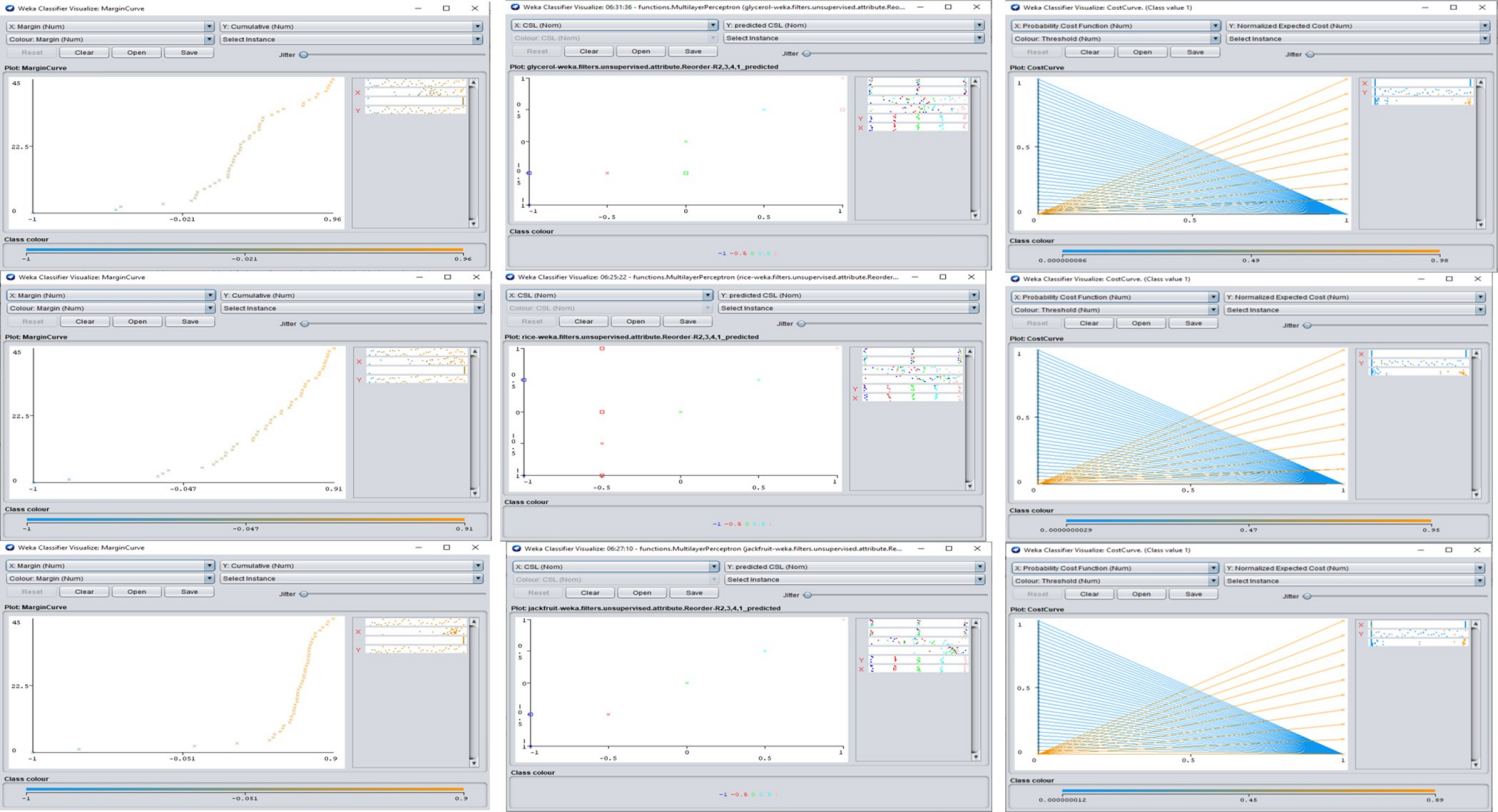
(a) Margin curve. (b) Classifier errors. (c) Cost curve; in glycerol, rice straw and jackfruit rind (from top to bottom).

In **Fig 1**, it is shown that the learning curve converges for the three carbon sources and the datasets are linearly separable. Thus, we can say that the one hidden layer is appropriate in the multilayer perceptron classifier and the model will not benefit from adding more data. Moreover, the prediction accuracy increases with the increase in training set size derived from the dataset and shows the invariability for model developed in multilayer perceptron. Thus, with the point of stability in the final two loss values, the model is considered to be a good fit. Moreover, continued training of a good fit model will likely lead to an over fit [35]. The problems such as over-fitting may be seen in multilayer perceptron during the maximum fit for the training data even when it fully converges [36]. To resolve this, cross validation can be performed on the dataset so as to train the model on all sets except one at each step [37]. **Fig 2(A)** shows the margin curve for the three carbon sources. The margin is defined as the difference between probability predicted for the actual class and the highest probability predicted for other classes [38]. A margin of value 1 means that the correct class was predicted with 100% confidence and a margin of value -1 means an incorrect class was predicted with similar confidence. As per the results, the classifier is confident in predicting the true classes. The central values of margin curve for glycerol, rice straw and jackfruit rind are -0.021, -0.047, and -0.051, respectively. **Fig 2(B)** represents the classifier errors plotted with class variable against the predicted class variable. The graph shows instances incorrectly classified with different colours for different class levels. **Fig 2(C)** represents the cost curve of classifier for the expected cost in three carbon sources. The cost curve plots the expected cost of using classifier against the probability cost function, which is a partisan type of *P*(+) and is at the same extremes: zero when *P*(+) = 0 and one when *P*(+) = 1 [39]. The cost curves serve as a valuable tool for assessing the performance of classification models while considering the balance between false positives and false negatives, which may incur different costs. It is denoted by *C*[+|−] the cost of predicting + when the instance is actually −, and the contrary by *C*[−|+]. The axis are described as

Normalisedexpectedcost=fn×Pc(+)+fp×(1−Pc(+))

Normalized Expected Cost (NEC) is integral to cost curve analysis and involves calculating the expected cost for different probability thresholds, where the threshold determines the model’s classification boundary. *fn* is the false negative rate, *Pc*(+) is the probability of a positive instance, *fp* is the false positive rate, and (1−*Pc*(+)) represents the probability of a negative instance. This normalization helps in ensuring that the expected cost is proportional to the likelihood of different outcomes and provides a balanced assessment of the model’s performance in terms of cost considerations. It is often used in situations where the costs associated with false positives and false negatives are not equal, allowing for a more tailored evaluation of the classification model’s impact on real-world applications. Probability Cost Function (PCF) is a mathematical representation or curve that illustrates the relationship between the probability of predicting a positive instance (*Pc*(+)) and the associated cost. It also represents the cost implications at different probability levels, helping to identify an optimal operating point that aligns with specific cost constraints or efficiency parameters.


$$Probability\;cost\;function=Pc(+)=\frac{P(+)C[-|+]}{P(+)C[-|+]+P(-)C[+|-]}$$

*Pc*(+) represents the probability of predicting a positive instance, *P*(+)*C*[−|+]) denotes the product of the probability of a positive instance (*P*(+)) and the cost associated with misclassifying a positive instance as negative (*C*[−|+]), *P*(+)*C*[−|+]+*P*(−)*C*[+|−] is the sum of the expected cost of false negatives (*P*(+)*C*[−|+]) and the expected cost of false positives (*P*(−)*C*[+|−]). The cost curve plots error rate as a function of *p*. (+) and error rate is the y-axis in the plot, *p*(+) is the x-axis [40]. The extreme values on the x-axis represent the situations where all the iterations to which the classifier will be applied are in the same class. If, *x* = *p*(+) = 0 means that all these iterations are negative, and if *x* = *p*(+) = 1 means they are all positive. If *x* = 0, a classifier’s overall error rate is simply its error rate on the negatives, since no positive iterations will be presented to the classifier. When x = 1 its overall error rate is its error rate on the positives. Joining the two points by a straight line plots its overall error rate as a function of *p*(+). This presents a single classifier which corresponds to straight line and shows how the performance varies as class distribution changes [41]. The cost curves are the plots with expected error against the probability of one of the classes. The diagonals show the performance of two extreme classifiers, The first predicting ‘+’ giving an expected error of one if the dataset contains no ‘+’ instances and zero if all instances are ‘+’. The second predicts ‘-’ which represents the contrary (always wrong) performance of classifier. A good classifier have low error rates with diagonals as close to the bottom as possible [42]. If P (+) is closer to the horizontal axis then the predictor has outperformed the classifier which always predicts ‘-’ and vice versa.

### Research gaps and future prospects

The current application of classifiers in predicting CSL concentration for microbial production processes has revealed appropriate research gaps and various prospects for future research. The identification of multilayer perceptron (MLP) as the optimal classifier with optimal results shows promising solutions to future scope, but the optimising novel classifiers could explore into emerging techniques and assess their applications in predicting CSL concentration (class variable). The current study primarily focuses on CSL concentration as the class variable. Future studies could broaden the scope by incorporating additional relevant features. Including a more extensive set of variables associated with microbial production processes could result in more nuanced and accurate classifiers, capturing the intricacies of the biological systems under investigation. This can be explored by adding more experimental variables, but would require even more lab trials in full factorial designs. To enhance the depth of understanding and the practical relevance of the classifiers developed, collaboration with experts from allied domains is essential. Integrating domain-specific knowledge can lead to classifiers with better alignment of microbial processes, achieving a synergistic relationship between data science and domain expertise. The study acknowledges the inherent complexity and black-box nature of MLPs. Future research can prioritize developing interpretable models or employing post-hoc interpretability techniques. This is crucial for gaining insights into the decision-making processes of classifiers, making the models more accessible and useful for stakeholders who may lack expertise in machine learning. While the current study provides valuable insights from an in-vitro setting, the application of classifiers to real-world microbial production environments is paramount. Future research should focus on the practical validation of models in industrial-scale processes, considering the dynamic and diverse conditions encountered in actual production scenarios. While sensitivity, specificity, accuracy, and ROC area are standard metrics, there is room for exploring additional performance metrics tailored to microbial production contexts. Metrics that specifically account for the challenges and intricacies of microbial processes could provide a more comprehensive evaluation of classifier performance. The study underscores the importance of data quality. Future investigations should aim for larger, more representative datasets while addressing potential biases. Implementing robust data augmentation techniques can mitigate challenges associated with data quantity and quality, enhancing the reliability of classifiers. Evaluating the robustness of the MLP classifier under diverse conditions is essential. Future studies can stress test the model by considering variations in experimental protocols, different carbon sources, and other relevant factors. Robust classifiers can ensure reliable predictions in scenarios beyond the specific conditions investigated in the current study. Future research can incorporate deeper biological insights into the modeling process. Understanding how specific biological factors influence the classifiers can lead to more accurate predictions. Models that align closely with the underlying microbial processes offer a more nuanced understanding of the intricate relationships within the system.

## Conclusions

This study is a comprehensive screening of classifiers for predicting CSL concentration based on in-vitro experimental data. The evaluation conducted uses various statistical measures, including sensitivity, specificity, accuracy, and area under the ROC curve. These measures played a crucial role in determining the most effective classifier for the model-building process. The class variable, CSL concentration, was used to assess the accuracy of different classifiers, with glycerol chosen as the standard carbon source and comparing it with organic carbon sources viz. rice straw and jackfruit rind for experimental design and modelling. The comparison of classifiers focused on pyruvate concentration, and the selected classifier was subsequently applied to other carbon sources for CSL concentration classification. The result analysis revealed that the multilayer perceptron (MLP) exhibited the highest accuracy (68.8889%) using 10X cross-validation, outperforming classifiers like simple logistic and IBk. Notably, MLP proved to be superior in terms of data mining accuracy, with neural networks generally showcasing more flexibility and outperforming logistic regression. This experimental comparison strongly supported the selection of MLP as the best-performing classifier among the alternatives. Following the classifier selection, MLP was applied to three distinct carbon sources—glycerol, rice straw, and jackfruit rind—to create models for predictive classification in microbial production. The model demonstrated high accuracy for each carbon source, with correctly classified instances ranging from 91.12% to 97.78%. Cohen’s Kappa statistic further confirmed the strength of the model, showing strong to almost perfect results for all three carbon sources. The low values of root mean squared error (RMSE) indicated the accuracy of the classifier in predicting CSL concentration. The application of the classifier model was extended to investigate overall accuracy, with precision, recall, and ROC area values considered. The results for the three carbon sources showcased outstanding performance, with ROC area values exceeding 0.9, indicating the model’s appropriateness for accurate predictions in the dataset. The weighted average precision and recall values further supported the robustness of the MLP classifier across all carbon sources. The versatility of MLP in practical applications for full factorial experimental design was highlighted through model validation. The learning curve analysis demonstrated that a single hidden layer was appropriate for the MLP classifier, and the model exhibited stability with increasing training set size. Additional analyses, including the margin curve, classifier errors, and cost curve, provided further insights into the classifier’s confidence and reliability. The classifier’s ability to predict true classes with confidence was evident in the margin curve, and the cost curve plotted error rates as a function of class distribution. However, the limitations of this study may be represented in the areas of generalisation of datasets (quantity and quality), extensive computational resource requirements, scale up and external validity using regression models, and model robustness due to experimental designs. Hence, the detailed statistical measures, model summary, and validation analyses collectively affirm the credibility and reliability of the MLP classifier for practical applications in experimental design and microbial production. This research paves the future scope for algorithmic advancements, feature engineering, hybrid model development, and most importantly interdisciplinary collaborations.

## Supporting information

S1 File Contains all the supporting tables and figures. (DOCX)
