# Supplementary material for: Evaluating classification tools for the prediction of in-vitro microbial pyruvate yield from organic carbon sources
Source: PLoS One. 2024 Jul 11;19(7):e0306987. doi: 10.1371/journal.pone.0306987 (PMC11239041; doi:10.1371/journal.pone.0306987)
Supplement: S1 File — (DOCX) [file pone.0306987.s001.docx]

**Evaluating classification tools for the prediction of *in-vitro* microbial pyruvate yield from organic carbon sources**

Manish Pant^a^*, Tanuja Pant^b*^

^a^IMS Engineering College, India

^b^Kumaun University, India

^*^Corresponding Author.


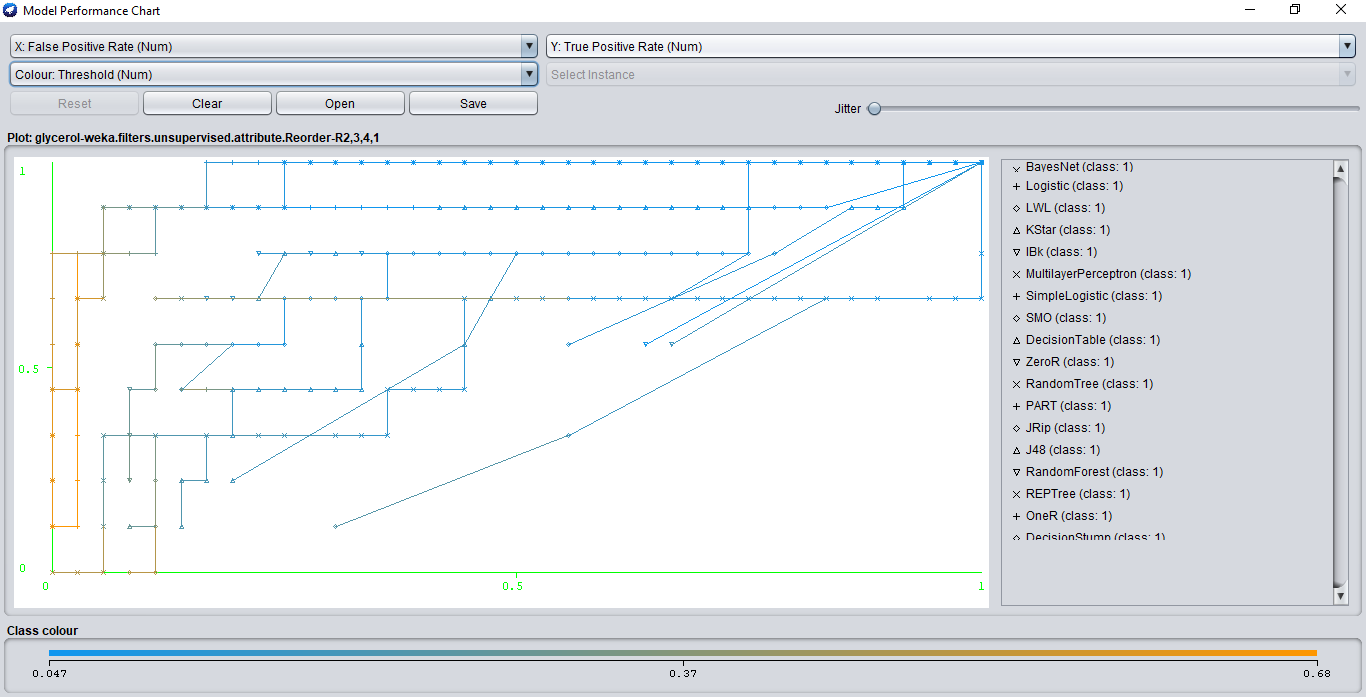


**Figure S1. Model performance chart: ROC curves for all possible classifiers**

**
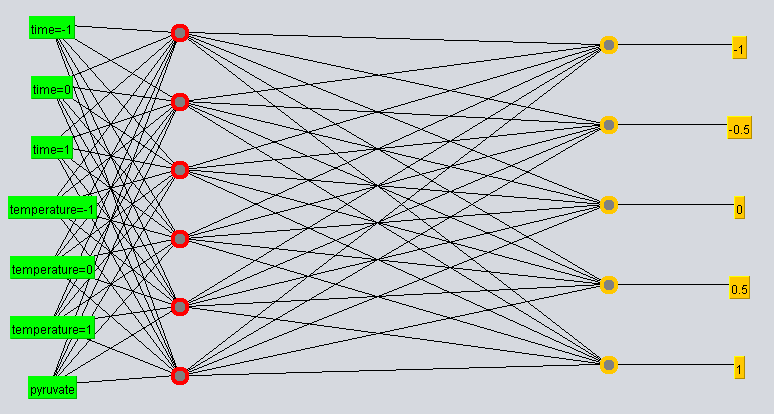
**

**Figure S2. Multilayer perceptron for the design model**

**
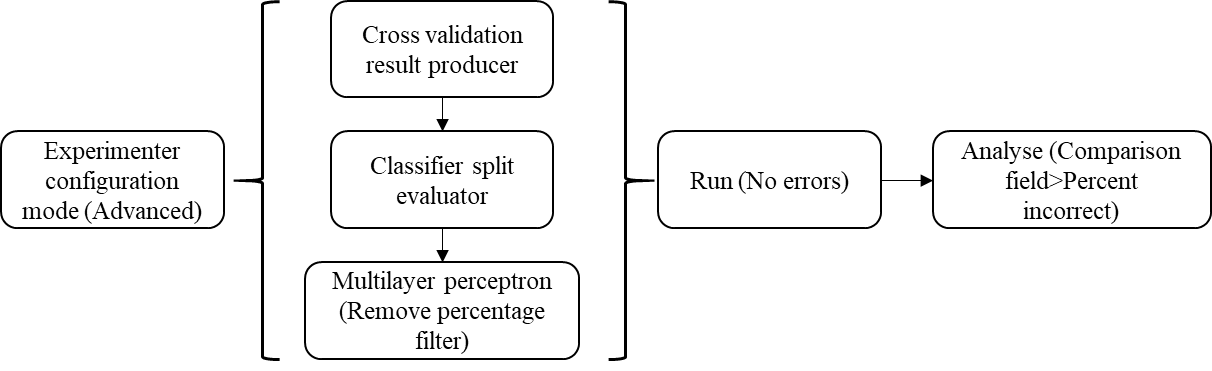
**

**Figure S3. Outflow for the comparison of classifiers**

**
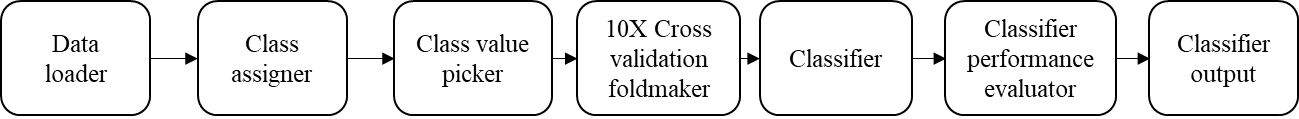
**

**Figure S4. Outflow for the learning curve**

**
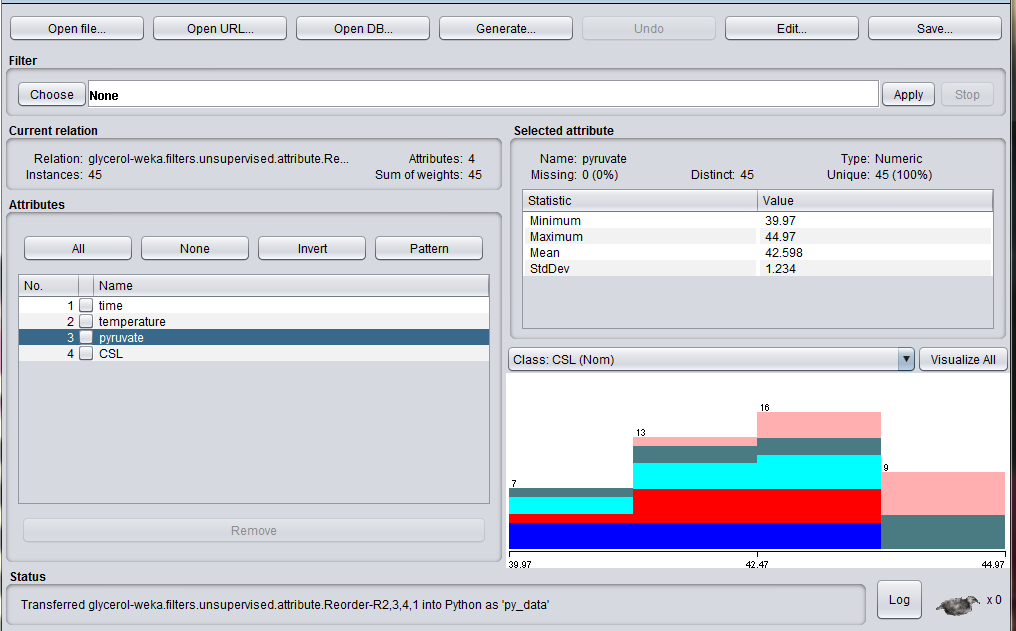

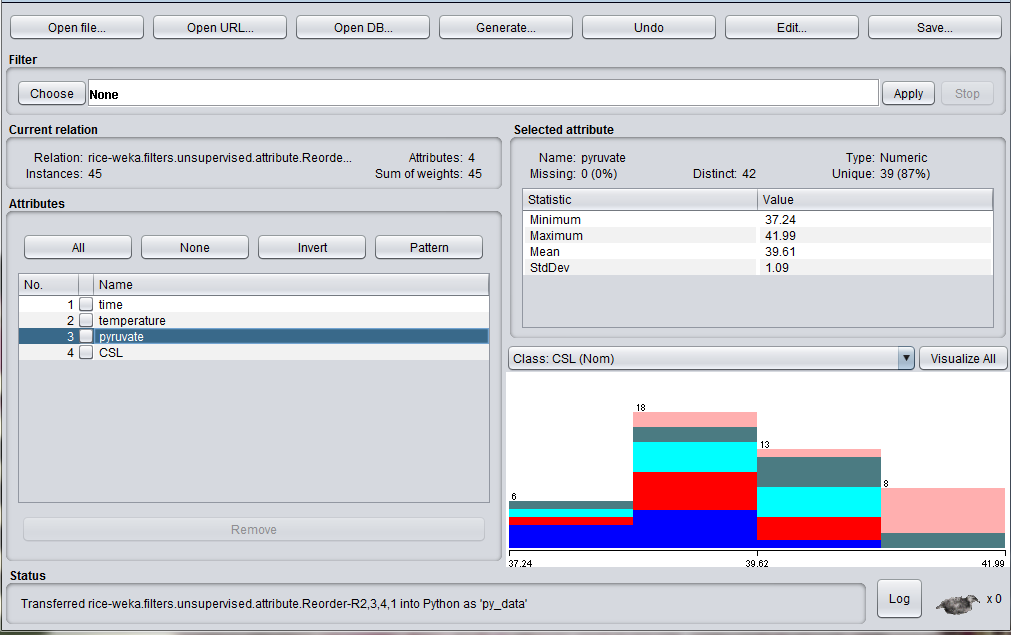
**

**
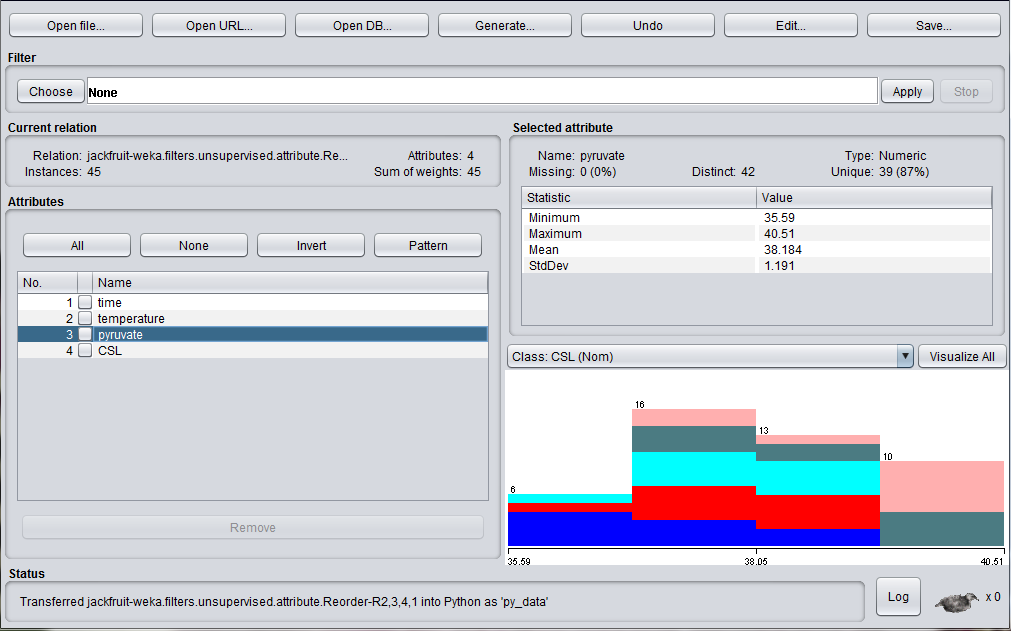
**

**Figure S5. Preprocess tab for the three carbon sources**

**Table S1. List of Symbols**

| **Sr. No.** | **Symbols** | **Full Form** |
| --- | --- | --- |
| 1. | f() | Function of |
| 2. | 10X | 10 times |
| 3. | % | Percentage |
| 4. | v/v | Volume per unit volume |
| 5. | $^$ | Raised to the power |
| 6. | h | Hour |
| 7. | °C | Degree centigrade |
| 8. | $\Sigma$ | Summation |
| 9. | $\kappa$ | Kappa |
| 10. | MLP | Multilayer Perceptron |
| 11. | CSL | Corn Steep Liquor |
| 12. | g | Gram |
| 13. | L | Litre |

**Table S2. Experimental design summary**

| **Run** | **F1** | **F2** | **F3** | **PC** | **Run** | **F1** | **F2** | **F3** | **PC** |
| --- | --- | --- | --- | --- | --- | --- | --- | --- | --- |
|  | **%v/v** | **h** | **℃** | **g/L** |  | **%v/v** | **h** | **℃** | **g/L** |
| 1 | 0 | 0 | 1 | 43.39 | 24 | -0.5 | -1 | 0 | 41.66 |
| 2 | 0 | 1 | 1 | 43.51 | 25 | -0.5 | -1 | -1 | 40.22 |
| 3 | -0.5 | 0 | -1 | 41.42 | 26 | 1 | 0 | 1 | 44.45 |
| 4 | -0.5 | 1 | 1 | 43.01 | 27 | 1 | -1 | -1 | 41.91 |
| 5 | -1 | -1 | 0 | 41.57 | 28 | -1 | 0 | 1 | 42.95 |
| 6 | 0.5 | -1 | 1 | 42.24 | 29 | -1 | 1 | 1 | 42.67 |
| 7 | -0.5 | -1 | 1 | 41.89 | 30 | 1 | -1 | 0 | 42.89 |
| 8 | -0.5 | 1 | 0 | 42.78 | 31 | -0.5 | 1 | -1 | 41.34 |
| 9 | -1 | 0 | 0 | 42.57 | 32 | 0.5 | -1 | -1 | 41.19 |
| 10 | 0 | 1 | -1 | 41.15 | 33 | 0.5 | 0 | -1 | 42.79 |
| 11 | -0.5 | 0 | 1 | 43.09 | 34 | 0 | 1 | 0 | 43.44 |
| 12 | 0.5 | 1 | -1 | 43.11 | 35 | 1 | 0 | -1 | 43.71 |
| 13 | 1 | -1 | 1 | 42.65 | 36 | 0 | -1 | -1 | 40.63 |
| 14 | 1 | 0 | 0 | 44.69 | 37 | 0 | 0 | 0 | 43.32 |
| 15 | -0.5 | 0 | 0 | 42.86 | 38 | 1 | 1 | -1 | 44.23 |
| 16 | -1 | -1 | 1 | 41.95 | 39 | 0 | -1 | 0 | 41.92 |
| 17 | 0 | 0 | -1 | 42.03 | 40 | 1 | 1 | 0 | 44.21 |
| 18 | 0.5 | 0 | 1 | 43.84 | 41 | 0 | -1 | 1 | 41.99 |
| 19 | 0.5 | -1 | 0 | 42.32 | 42 | 0.5 | 1 | 1 | 44.16 |
| 20 | 0.5 | 1 | 0 | 44.34 | 43 | 0.5 | 0 | 0 | 43.92 |
| 21 | -1 | 0 | -1 | 40.97 | 44 | 1 | 1 | 1 | 44.97 |
| 22 | -1 | -1 | -1 | 39.97 | 45 | -1 | 1 | 0 | 42.29 |
| 23 | -1 | 1 | -1 | 40.69 |  |  |  |  |  |

| **Table S3. Detailed accuracy by class for glycerol** | | | | | | | | | |
| --- | --- | --- | --- | --- | --- | --- | --- | --- | --- |
|  | TP Rate | FP Rate | Precision | Recall | F-measure | MCC | ROC Area | PRC Area | Class |
|  | 0.889 | 0.000 | 1.000 | 0.889 | 0.941 | 0.930 | 0.994 | 0.980 | -1 |
|  | 1.000 | 0.056 | 0.818 | 1.000 | 0.900 | 0.879 | 0.966 | 0.769 | -0.5 |
|  | 0.889 | 0.000 | 1.000 | 0.889 | 0.941 | 0.930 | 0.944 | 0.907 | 0 |
|  | 1.000 | 0.028 | 0.900 | 1.000 | 0.947 | 0.935 | 0.991 | 0.963 | 0.5 |
|  | 0.889 | 0.000 | 1.000 | 0.889 | 0.941 | 0.930 | 0.997 | 0.989 | 1 |
| **Weighted Avg.** | 0.933 | 0.017 | 0.944 | 0.933 | 0.934 | 0.921 | 0.978 | 0.921 |  |
| **Table S4. Detailed accuracy by class for rice straw** | | | | | | | | | |
|  | TP Rate | FP Rate | Precision | Recall | F-measure | MCC | ROC Area | PRC Area | Class |
|  | 0.889 | 0.028 | 0.889 | 0.889 | 0.889 | 0.861 | 0.981 | 0.948 | -1 |
|  | 0.667 | 0.000 | 1.000 | 0.667 | 0.800 | 0.784 | 0.858 | 0.818 | -0.5 |
|  | 1.000 | 0.028 | 0.900 | 1.000 | 0.947 | 0.935 | 1.000 | 1.000 | 0 |
|  | 1.000 | 0.028 | 0.900 | 1.000 | 0.947 | 0.935 | 1.000 | 1.000 | 0.5 |
|  | 1.000 | 0.028 | 0.900 | 1.000 | 0.947 | 0.935 | 0.997 | 0.989 | 1 |
| **Weighted Avg.** | 0.911 | 0.022 | 0.918 | 0.911 | 0.906 | 0.890 | 0.967 | 0.951 |  |

| **Table S5. Detailed accuracy by class for jackfruit rind** | | | | | | | | | |
| --- | --- | --- | --- | --- | --- | --- | --- | --- | --- |
|  | TP Rate | FP Rate | Precision | Recall | F-measure | MCC | ROC Area | PRC Area | Class |
|  | 0.889 | 0.000 | 1.000 | 0.889 | 0.941 | 0.930 | 0.972 | 0.944 | -1 |
|  | 1.000 | 0.028 | 0.900 | 1.000 | 0.947 | 0.935 | 0.997 | 0.989 | -0.5 |
|  | 1.000 | 0.000 | 1.000 | 1.000 | 1.000 | 1.000 | 1.000 | 1.000 | 0 |
|  | 1.000 | 0.000 | 1.000 | 1.000 | 1.000 | 1.000 | 1.000 | 1.000 | 0.5 |
|  | 1.000 | 0.000 | 1.000 | 1.000 | 1.000 | 1.000 | 1.000 | 1.000 | 1 |
| **Weighted Avg.** | 0.978 | 0.006 | 0.980 | 0.978 | 0.978 | 0.973 | 0.994 | 0.987 |  |

| **Table S6. Confusion matrix for the design model of glycerol** | | | | | | | | | | |
| --- | --- | --- | --- | --- | --- | --- | --- | --- | --- | --- |
| A | B | | C | | d | | e | | Classified as | |
| 8 | 1 | | 0 | | 0 | | 0 | | a = -1 | |
| 0 | 9 | | 0 | | 0 | | 0 | | b = -0.5 | |
| 0 | 1 | | 8 | | 0 | | 0 | | c = 0 | |
| 0 | 0 | | 0 | | 9 | | 0 | | d = 0.5 | |
| 0 | 0 | | 0 | | 1 | | 8 | | e = 1 | |
| **Table S7. Confusion matrix for the design model of rice straw** | | | | | | | | | | |
| A | B | | C | | d | | e | | Classified as | |
| 8 | 0 | | 0 | | 1 | | 0 | | a = -1 | |
| 1 | 6 | | 1 | | 0 | | 1 | | b = -0.5 | |
| 0 | 0 | | 9 | | 0 | | 0 | | c = 0 | |
| 0 | 0 | | 0 | | 9 | | 0 | | d = 0.5 | |
| 0 | 0 | | 0 | | 0 | | 9 | | e = 1 | |
| **Table S8. Confusion matrix for the design model of jackfruit rind** | | | | | | | | | | |
| A | | B | | C | | d | | e | | Classified as |
| 8 | | 1 | | 0 | | 0 | | 0 | | a = -1 |
| 0 | | 9 | | 0 | | 0 | | 0 | | b = -0.5 |
| 0 | | 0 | | 9 | | 0 | | 0 | | c = 0 |
| 0 | | 0 | | 0 | | 9 | | 0 | | d = 0.5 |
| 0 | | 0 | | 0 | | 0 | | 9 | | e = 1 |
